# Supplementary material for: I struggle with your fidgeting: A qualitative study of the personal and social impacts of misokinesia
Source: PLoS One. 2024 Dec 4;19(12):e0313169. doi: 10.1371/journal.pone.0313169 (PMC11616835; doi:10.1371/journal.pone.0313169)
Supplement: S1 Table — (DOCX) [file pone.0313169.s001.docx]

S1 Table

Raw misokinesia (MkAQ), misophonia (MpAQ), and anxiety (STAI) scores reported in this manuscript.

| ID: | MpAQ | MkAQ | state_sum | trait_anxiety |
| --- | --- | --- | --- | --- |
| 1 | 41 | 52 | 24 | 46 |
| 2 | 44 | 45 | 55 | 57 |
| 3 | 63 | 62 | 73 | 73 |
| 4 | 52 | 54 | 59 | 49 |
| 5 | 30 | 44 | 68 | 77 |
| 6 | 63 | 63 | 60 | 66 |
| 7 | 2 | 4 | 23 | 27 |
| 8 | 54 | 45 | 44 | 53 |
| 9 | 28 | 28 | 45 | 56 |
| 13 | 63 | 58 | 36 | 38 |
| 14 | 18 | 16 | 52 | 53 |
| 16 | 6 | 10 | 46 | 65 |
| 17 | 60 | 48 | 38 | 61 |
| 18 | 40 | 52 | 56 | 62 |
| 19 | 55 | 47 | 38 | 45 |
| 21 | 4 | 19 | 30 | 36 |
| 22 | 35 | 7 | 40 | 54 |
| 23 | 7 | 42 | 27 |  |
| 24 | 43 | 35 | 52 | 60 |
| 25 | 55 | 55 | 46 | 47 |
| 28 | 62 | 56 | 27 | 29 |
